# Effects of Red Blood Cell Transfusions on Distant Metastases of Oral Squamous Cell Carcinomas

**DOI:** 10.3390/cancers14010138

**Published:** 2021-12-28

**Authors:** Leonard Simon Brandenburg, Marc Christian Metzger, Philipp Poxleitner, Pit Jacob Voss, Kirstin Vach, Johannes Hell, Konstantin Hasel, Julia Vera Weingart, Steffen Jochen Schwarz, Michael Andreas Ermer

**Affiliations:** 1 Department of Oral and Maxillofacial Surgery, Clinic, Medical Center—University of Freiburg, Faculty of Medicine, University of Freiburg, Hugstetterstr. 55, 79106 Freiburg, Germany; marc.metzger@uniklinik-freiburg.de (M.C.M.); philipp.poxleitner@uniklinik-freiburg.de (P.P.); pit.voss@uniklinik-freiburg.de (P.J.V.); konstantin.hasel@uniklinik-freiburg.de (K.H.); julia.weingart@uniklinik-freiburg.de (J.V.W.); steffen.schwarz@uniklinik-freiburg.de (S.J.S.); michael.ermer@uniklinik-freiburg.de (M.A.E.); 2 Institute of Medical Biometry and Statistics, Faculty of Medicine and Medical Center, University of Freiburg, Zinkmattenstr. 6A, 79108 Freiburg, Germany; kv@imbi.uni-freiburg.de; 3 Department of Anesthesiology and Critical Care, Clinic, Medical Center—University of Freiburg, Faculty of Medicine, University of Freiburg, Hugstetterstr. 55, 79106 Freiburg, Germany; johannes.hell@uniklinik-freiburg.de

**Keywords:** red blood cell transfusion, oral squamous cell carcinoma, distant metastasis, risk factor

## Abstract

**Simple Summary:**

Patients with distant metastasis of oral squamous cell carcinoma should be identified at an early stage of disease. In this study, we investigated if patients who received red blood cell transfusions are at risk for the development of distant metastasis. A positive correlation was found between RBC transfusion (HR = 2.42) and the occurrence of M+ in a multivariate regression model. Therefore, the administration of RBC can be considered as an independent prognostic factor and special attention should be paid to its detrimental effects in the perioperative management of OSCC patients.

**Abstract:**

There is no consensus on the effect of red blood cell (RBC) transfusions on patients with oral squamous cell carcinoma (OSCC). The aim of this study was to investigate the association between RBC administration and the occurrence of distant metastases (M+) after surgical treatment of OSCC. All medical records of patients who underwent primary surgery for OSCC in our department (2003–2019) were analyzed retrospectively (*n* = 609). Chi and Cox regression models were used to analyze the influence of transfusion on the development of M+, and survival rates. Kaplan–Meier curves were used for graphical presentation. A multitude of patient-specific factors showed a statistical impact in univariate analysis (transfusion, age, gender, diabetes, pT, pN, L, V, Pn, G, UICC, adjuvant therapy, free microvascular transplant, preoperative hemoglobin level). Transfusion status and pN stage were the only variables that showed a significant correlation to M+ in the multivariate Cox model. The hazard ratios for the occurrence of M+ were 2.42 for RBC transfusions and 2.99 for pN+. Administration of RBC transfusions was identified as a significant prognostic parameter for the occurrence of distant metastases after surgical treatment of OSCC. Hence, the administration of RBC transfusions should be considered carefully in the perioperative management.

## 1. Introduction

No drug can compensate for the lack of erythrocytes due to bleeding or disease, making human red blood cells (RBC) indispensable in medicine [1]. Despite its life-saving potential in the treatment of anemia, detrimental effects of RBC transfusions are described. Besides the risk for infection and allergic or hemolytic reactions, a transient immunomodulation caused by RBC transfusions was confirmed [2,3,4].

Opelz first observed the immunomodulatory effect of RBC transfusions in the early 1970′s. He discovered increased survival rates of kidney transplants in correlation with RBC transfusion due to induction of immune unresponsiveness [5,6]. Further studies in this field revealed the underlying mechanisms on cellular [7,8,9] and humoral levels [10,11,12], which are known as transfusion-related immunomodulation (TRIM) since the early 1990′s [3]. These findings led to a paradigm shift in the management of anemic patients [13]. In the early 2000s, leukocyte depletion was introduced almost universally as a measure to reduce transfusion-associated complications [14,15]. In general, the use of RBC transfusions is evaluated more critically to avoid transfusion-related complications [16].

In the field of surgery, certain complications are associated with RBC transfusion. Due to TRIM, a higher rate of surgical site infections is observed [17,18,19]. In the current literature, the effect of RBC transfusions on the overall survival (OS) and tumor-free survival (TFS) of cancer patients is discussed controversially. For malignancies of the colon [20], lung [21], and stomach [22] significant correlations are found between the administration of RBC transfusions and decreased survival rates.

Oral cancer affects 4.0 out of 100,000 patients worldwide and is the 6th most common type of cancer [23]. Oral squamous cell carcinoma (OSCC) is the most frequent type of cancer in the oral cavity [23]. Local resection with cervical lymph-node dissection is the primary treatment of choice in curative intention. Various drugs and treatments have been proposed to manage this condition, but current alternative options are not satisfactory, having only a moderate impact on unresectable cancers [24,25]. Despite the importance of OSCC in global health, there is little evidence on the role of RBC transfusions in relation to the long-term outcome of patients with OSCC. Several authors investigated the effect of RBC transfusions on OS and TFS in patients with OSCC, however, they came to different conclusions [26]: While some authors could not prove the adverse effect of RBC transfusions, [27,28,29,30,31] other studies report significantly shorter OS and TFS in transfused OSCC patients [30,31].

As a result of the restrictive transfusion regimen, only a small portion of OSCC patients nowadays receive RBCs in perioperative management [13]. These patients are often most severely affected by the OSCC and have multiple comorbidities, which make them susceptible to anemia. Hence, not only the transfusion status, but many confounding factors influence the long-term outcome of these patients [32]. The aim of this study was to assess the correlation between the occurrence of distant metastases (M+) of OSCC in dependence on RBC transfusions. Survival rates (OS and TFS) were investigated as secondary outcome variables. The hypothesis of this study was that the administration of RBCs in the perioperative care of OSCC patients significantly increases the probability of the development of M+.

## 2. Materials and Methods

This retrospective cohort study includes patients treated for OSCC between 2003 and 2019 from a single tertiary center (Clinic of Oral and Maxillofacial Surgery, Medical Center—University of Freiburg, Germany). The study was conducted in accordance with the Helsinki Declaration and the Ethics Committee of the University of Freiburg (No. 127/15) approved the study protocol.

The main prognosis factor investigated in this study was the transfusion status. The amount of transfused RBC units during surgery and within the first 14 days after surgery was counted and patients were categorized into three groups, receiving 0, 1–3, or >3 RBC units. The aim of the study was to investigate the effect of RBC administration on the outcome variables.

The primary outcome variable in this study was the development of M+ after surgery. Secondary outcome variables were the event of death (to investigate OS) or relapse of OSCC (to investigate TFS).

### 2.1. Study Group

Patients treated with primary surgery for OSCC at our institution had to meet the following criteria in order to be included in the study: minimum age of 18 years, cancer-free resection margins (R0), surgical treatment, and post-operative follow-up of at least 12 months was performed at our department, consistent documentation was available via the electronic patient charts. All consecutive cases with primary tumor resections were included without regard to reconstruction procedures.

Malignancies other than OSCC, secondary surgical cases for relapsed OSCC, evidence of distant metastasis at initial diagnosis, and neo-adjuvant therapy prior to surgery led to exclusion.

Administered RBC units were obtained from the Department for Transfusion Medicine of the University Medical Centre in Freiburg and were leukocyte depleted.

### 2.2. Data Acquisition

The collected data were provided from the Clinic of Oral and Maxillofacial Surgery and related departments of the Medical Center—University of Freiburg, Germany. Electronic patient charts were reviewed by one investigator (KH) and were arranged in a Microsoft Excel spreadsheet (Microsoft Excel^®^ Version 16.0, Microsoft Corporation, Albuquerque, NM, USA). Variables not to be determined by thorough chart review were labeled as missing data.

Patient-specific factors concerning the state of health and therapy were collected to identify possible confounding variables. Demographic features (age and gender), carcinogenic substance abuse, TNM classification according to UICC 2016 [33], histopathological grading, tumor localization, use of microvascular transplant for reconstruction, preoperative hemoglobin value, amount of transfused RBC units during surgery and within the first 14 days after surgery, and consecutive adjuvant therapy were documented. Follow-up examinations were performed in our outpatient clinic according to the German clinical practice guideline [34]. Events of local and regional recurrence—as well as development of metachronous distant metastasis—were recorded.

To improve data quality on recurrence, distant metastasis, and survival, the Comprehensive Cancer Center of the University Hospital Freiburg (CCCF) was queried whether other healthcare providers reported relapse, development of M+, or death of the included patients to the central cancer registry. Subsequently, follow-up, time to recurrence, time to distant metastasis, and time to death were calculated.

Histopathologic specifications of the OSCC were determined by the Institute for Surgical Pathology of the University Medical Centre in Freiburg.

### 2.3. Statistical Analysis

Due to the retrospective character of the study, there was no sample size calculation, instead, all patients who fulfilled the mentioned inclusion criteria were analyzed. Assuming a prevalence of 30% transfusions and a 10-year survival probability of 70%, a hazard ratio of 0.5 can be found with 90% power using 500 patients.

For the analyses, the statistics program STATA (StataCorp LT, College Station, TX, USA, Version 16.1) was used. For descriptive analyses mean, standard deviations, and relative frequencies were computed.

To evaluate possible correlations of other patient-specific features regarding health and the therapy with each of the three outcome variables (M+, OS, and TFS), univariate Cox regression analyses were performed; to check an association of patient-specific factors and transfusion status, Fisher’s exact test was used.

A Cox regression model adjusting for age, gender, pT, pN, free microvascular grafts, and preoperative hemoglobin was used to analyze the influence of transfusion on the outcome variables (multivariate analysis). For subsequent pairwise comparisons, corrections were made according to Scheffe’s method for multiple testing.

Kaplan–Meier curves were used for graphical presentation. The significance level was set to *p* = 0.05.

## 3. Results

### 3.1. Study Group Characteristics

A total of 609 patients received primary surgery of OSCC between 2003 and 2019 at the Clinic of Oral and Maxillofacial Surgery, Medical Center—University of Freiburg, Germany. 21 patients were excluded because of R1-resection status after primary surgery. Five hundred and eighty-eight patients (334 men and 254 women) were included with an overall mean age of 64.0 ± 12.5 years. RBC units were transfused in 152 (28.9%) cases in total. 102 patients received 1–3 RBC units, and 50 patients received >3 RBC units. Three hundred and eighty-eight (75.2%) of all RBC units were administered in the first 72 h after surgery. Transfusion rates of men (27.9%) and women (23.2%) were comparable. The median follow-up time was 5.5 ± 4.5 years.

Table 1 shows the patient characteristics of the study group in relation to the transfusion status. The administration of RBC transfusions is significantly associated with the following variables: nicotine, alcohol, localization, adjuvant therapy, use of microvascular transplants, pT-, pN-, L-, and UICC-classification.

In our study population, M+ was reported in 74 (12.6%) patients with four patients presenting synchronous M+ in multiple locations, adding up to 80 locations. The predominant location of M+ were the lungs (*n* = 41, 51.1%), followed by deep soft tissue metastases (*n* = 15, 18.8%), liver (*n* = 7, 8.8%), non-cervical lymph nodes (*n* = 6, 7.5% (4 infraclavicular, 1 thoracic, 1 axillary)), skeleton (*n* = 4, 5.0%), brain (*n* = 3, 3.8%), adrenal gland (*n* = 2, 2.5%) and skin (*n* = 2, 2.5%).

Death and relapse occurred in 321 (54.6%) and 241 (40.0%) cases respectively. Mean time until the occurrence of M+ was 4.92 ± 4.36 years. Mean overall survival and tumor-free survival were 5.31 ± 4.34 years and 4.24 ± 4.11 years, respectively. A statistically significant correlation of the transfusion status (yes/no) was observed for M+ and OS.

Thirty-three (44.6%) patients who were affected by M+ received RBC transfusions. RBCs were administered to patients who died or suffered a relapse in 73 (22.8%) and 59 (24.5%) cases, respectively. Patients with an occurrence of M+, death, or relapse received RBC transfusions significantly more often (*p* < 0.01).

The mean time from first tumor surgery until the occurrence of M+ in all 74 patient cases was 28.3 ± 35.8 months. Thirty-eight patients developed a local relapse of OSCC prior to M+ diagnosis. They received a local recurrence surgery 20.3 ± 24.1 months after the primary tumor resection. In these patients, the mean time between recurrence surgery and M+ was 33.7 ± 40.0 months. Thirty-four patients developed M+ without prior local relapse in the observational period. In these cases, the mean time from initial tumor resection until the occurrence of M+ was 23.5 ± 31.0 months. Two patients developed M+ first and were later diagnosed with a local recurrence. In these two patients, mean time from tumor resection until M+ was 7.1 months ± 6 days.

UICC classification could be determined for all patients. The most frequent UICC classification diagnosed was I (UICC I = 39.8%, UICC II = 19.6%, UICC III = 15.9%, UICC IV = 24.7%). The localization of OSCC was distributed as follows: 45.4% floor of mouth and lower jaw (*n* = 267), 25.2% tongue (n = 148), 11.05% upper jaw (*n* = 65), 8.2% oral cheek and lip (*n* = 48), 6.8% multilocular (*n* = 40) and 3.2% oropharynx (*n* = 19). 214 (36.4%) patients required reconstruction with a free microvascular transplant. Preoperative hemoglobin level was less than 12 g/dL in 67 (11.4%) of all patients. Adjuvant radiation or radio-chemotherapy was administered in 181 (30.8%) cases. Two hundred and nine (35.6%) patients reported regular consumption of alcohol and 285 (48.5%) patients reported to be smokers.

### 3.2. Univariate Analysis

A Cox proportional hazards regression analysis was performed for all the study variables individually regarding M+, OS, and TFS. The following variables are significantly correlated with the occurrence of M+: transfusion, age, gender, pT, pN, L, V, Pn, G, UICC, adjuvant therapy, free microvascular transplant, preoperative hemoglobin. Shorter OS was significantly correlated with transfusion, age, gender, pT, pN, localization, L, V, Pn, G, UICC, adjuvant therapy, microvascular transplant, and preoperative hemoglobin. TFS was significantly shorter in patients in dependence of transfusion status, pT, pN, localization, L, Pn, G, UICC, adjuvant therapy, free microvascular transplant, and preoperative hemoglobin. Table 2 shows the results of the univariate analysis.

### 3.3. Multivariate Analysis

A Cox regression model with the additional factors age, gender, pT, pN, microvascular transplant, and preoperative hemoglobin was used to analyze the influence of RBC transfusion on the outcome variables. RBC transfusion and pN had a significant correlation with M+ when adjusted for age, gender, pT, microvascular transplant, and preoperative hemoglobin value. Shorter OS was significantly correlated with age, gender, pN, and the use of a free microvascular transplant in multivariate analysis. TFS only showed a significant correlation with age, pT, and pN > 2 in the multivariate analysis. Table 3 summarizes the results of the performed multivariate Cox regression analysis.

## 4. Discussion

**Figure 1:**
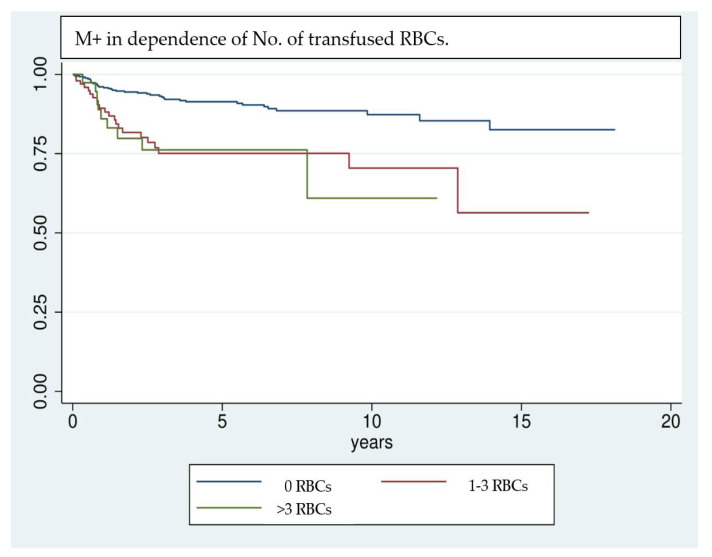
Kaplan–Meier analysis of the development of M+ in dependence of the number of transfused RBC units. Patients who did not receive RBC transfusions in the perioperative stage show a comparatively lower rate of M+. Especially in the first 3 years after surgery patients who received RBC transfusions tend to develop M+ more frequently.

**Figure 2:**
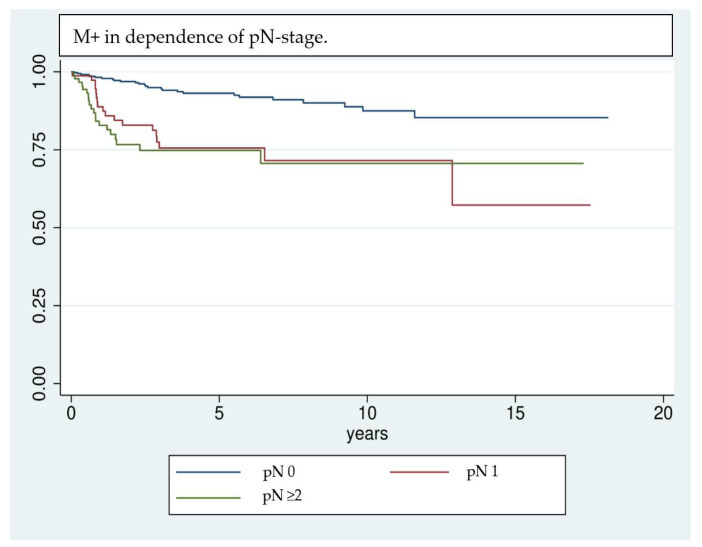
Kaplan–Meier analysis of the development of M+ in dependence of the pN stage. Patients who had lymph node invasion at the time of initial diagnosis are more likely to develop distant metastases in the further course of events.

This study found a significant correlation between the development of M+ and the administration of RBC transfusions. While 44.6% of all patients who developed M+ received RBC transfusions, only 24.6% of patients who did not develop M+ received transfusions perioperatively. The correlation of RBC transfusions with M+ was statistically significant in univariate analysis (*p* < 0.001). As M+ also showed a significant correlation to a multitude of further patient-specific features regarding health and therapy (pT-, pN-, L-, V-, Pn-, and UICC classification, grading, adjuvant therapy, use of free vascularized grafts (tx), and preoperative hemoglobin value) a multivariate Cox regression model was built to adjust for the effect of confounding variables. In this model, RBC transfusions (Figure 1) and pN stage (Figure 2) showed a significant correlation (see Table 3). Hence, RBC transfusions (Figure 1) and pN stage (Figure 2) proved to be independent risk factors for M+ even when adjusted for the main confounding factors. While lymph node status was already found to be an independent prognostic factor in previous studies [35,36,37,38], this is the first study to report RBC transfusions as a significant risk factor for M+. The hazard ratio (HR) of developing M+ is 2.42 times higher for transfused patients compared to non-transfused patients. The pN status comes with an even higher HR for the development of M+ (pN1 = 2.99, pN ≥ 2 = 3.37). Therefore, the transfusion state is the second important prognosis factor for the development of M+ among all reported factors in this study. Based on these results, restrictive transfusion regimens should be applied in the surgical treatment of OSCC and may decrease the likelihood of metastatic spread of OSCC.

**Figure 3:**
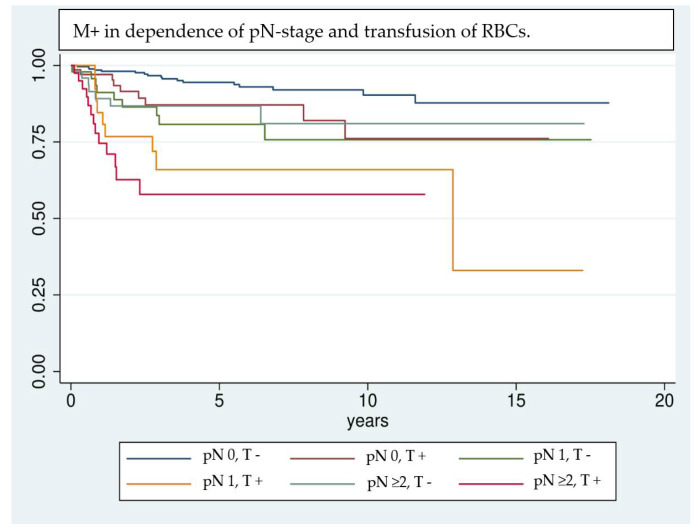
Comparison of patients according to their transfusion status (T +/−) and pN stage (pN 0, 1, or >= 2) using Kaplan–Meier analysis. In patients with lymph node invasion and transfusion the detrimental effects on the development of M+ are combined and lead to a remarkable worsening of the long-term outcome (pN0, T+ [red], pN1, T+ [yellow] and pN >= 2, T+ [pink]). It is notable that patients with transfusion but without lymph node invasion (red curve) show a comparable trend in the development of M+ as non-transfused patients with lymph-node invasion (light blue and green curve).

**Figure 4:**
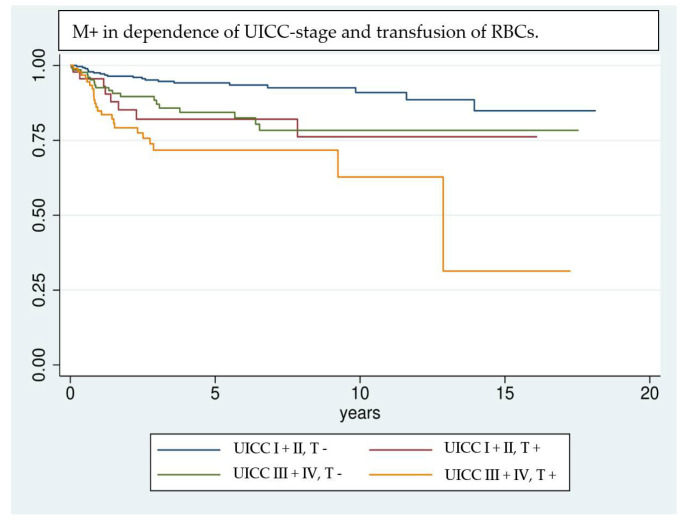
Comparison of patients according to their transfusion status (T +/−) and UICC classification UICC I + II vs. UICC III + IV) using Kaplan–Meier analysis. Patients with advanced tumor disease (UICC III + IV) but without RBC transfusion show a comparable trend in developing M+ as patients who are diagnosed in an earlier stage of disease (UICC I + II) but received transfusions in the perioperative management.

Even though the results are highly significant, it should be noted that results achieved by Cox regression models are highly dependent on the implemented variables [39]; e.g., the UICC classification was not included in multivariate analysis, because of interfering effects when adjusting for pT- and pN classification. Nevertheless, UICC classification has a significant effect on univariate analysis and shows a clear trend of being correlated with a higher risk of M+. Due to interference of some factors investigated in univariate analysis, only a selection could be implemented in the multivariate analysis. Factors that are unmodifiable (gender, age) or inherently linked with the tumor stage and show significance in univariate analysis (pT, pN, free Tx, preoperative hemoglobin value) are included in the regression model. This allows the investigation of factors most likely to affect the outcome variable, however, it can overestimate their influence [39]. Kaplan–Meier survival analysis (Figure 1, Figure 2 and Figure 3) shows that RBCs increase the likelihood of early development of M+. Remarkably, non-transfused patients with pN stage >0 develop M+ less frequently compared to patients with pN stage 0 who received transfusions (Figure 3). Likewise, when comparing patients with high UICC classifications (UICC III + IV) but without transfusion, longer latencies until the occurrence of M+ were found in comparison to patients with low UICC classifications (UICC I + II) but positive transfusion status (Figure 4).

While there is a plethora of literature that investigates the correlation of patient-specific features regarding health and therapy with the development of M+ in patients with OSCC [35,36,37,38,40,41], there is no study evaluating RBC transfusions as an independent factor. A recent study identified the incidence of primary intraosseous carcinoma of the mandible and cervical lymph node status as independent risk factors for the development of M+ in OSCC patients [37]. In comparison, our study did not reveal any correlations between the localization of the tumor and the occurrence of M+, but it also identified the pN stage as one of the most important risk factors. A strong correlation between nodal status and the occurrence of M+ was confirmed previously [35,36,37,38,40]. Even studies investigating head and neck squamous cell carcinoma, including carcinomas of the pharynx and larynx, confirmed this correlation [36,38]. Other authors postulated clinical lymph node status [35], extracapsular spread [37,38,40], or locoregional control [40] as independent prognostic factors and found them to be statistically significant. The important role of cervical lymph nodes in the treatment of OSCC and head and neck squamous cell carcinomas (HNSCC) could be explained by the higher amount of disseminated and circulating tumor cells due to extranodal expansion [41].

The distribution of M+ in OSCC patients was described similarly in previous studies [35,36,37,38,40], with the lung being the most frequent localization.

Studies previously conducted in our Clinic for Oral and Maxillofacial Surgery, Medical Center—the University of Freiburg already analyzed OS and TFS [24]. Since the data presented in the present study consists of an updated study group containing all patients recently operated in our department analyses regarding OS and TFS were repeated as previously described. This provides an overview regarding the long-term outcome of our study group while also allowing a comparison with other studies, which did not investigate the development of M+ [27,28,29,30,31]. In previous studies, a transfusion rate of 13–82% was reported. Death of any cause was observed in 26–57% of all patients and recurrence of OSCC occurred in 19–49% of patients [27,28,29,30,31]. The results of the present study lie within the stated range, with a mortality rate of 54% of any cause and a recurrence rate of 40%. With a transfusion rate of 29%, a restrained transfusion regime was followed in our study group. Low transfusion rates were achieved especially in more recent studies [28,29]. When comparing the statistical analyses of the correlation of RBC transfusions with relapse or death, similar results were achieved compared to previous studies [27,28,29,30,31]: While univariate analysis shows a significant correlation between transfusion status and early death or relapse (see Table 2), the multivariate analysis yields no significant results regarding the transfusion state (see Table 3). Many studies found a univariate significant correlation between RBC transfusion and survival rate, yet only a few could confirm this correlation after adjusting for confounding factors [30,31]. Notably, a high transfusion rate was reported (77–82%) in these studies.

In most studies, it remains unknown if patients suffered death because of OSCC or because of other causes. Therefore, most studies investigating survival rates are somehow biased. The role of other confounding variables appears to be statistically inseparable from the effect of RBC transfusions on patients’ survival [26]. This may explain why multivariate analyses in prior studies were mostly statistically insignificant. In contrast, the assessment of M+ could reveal the true effect of RBC transfusions on the oncologic outcome more properly. By investigating the development of M+ an unambiguously cancer-related outcome variable is assessed, which is not biased by death because of systemic diseases (e.g., delirium, hemodynamic complications, etc.). Nevertheless, the correlation of the patients’ health condition and the susceptibility for anemia and tumor-related complications must not be ignored when interpreting the results of the present study.

The immunosuppressive potency of RBC transfusions is undergoing extensive research. Various mechanisms are being described and discussed, such as the presence of residual leukocytes or apoptotic cells, potentially bioactive molecules as cytokines, active growth factors, or serum proteins and cell-derived extracellular vesicles [42,43]. Circulating tumor cells (CTC) are present in the bloodstream in the perioperative cancer surgery setting in numerous entities and usually correlate with tumor size, nodal infiltration, or metastatic spread [44,45] and can be likewise detected in the bone marrow of OSCC patients [46]. This condition is the foundation for various liquid biopsy methods aiming at early detection as well as for monitoring the course and creating prediction models of malignant diseases [47,48,49].

In the context of the highly significant and previously described well-understood principles of TRIM [3], a disadvantageous effect of RBC transfusions on metastatic spread is very likely and should be considered in the perioperative management of cancer patients. Recent publications do not only report immuno-modulatory effects but also propose pro-inflammatory effects caused by RBC transfusions. Allogeneic white blood cells but also bioactive lipids and other soluble mediators induce an undesirable pro-tumor inflammatory response [3,50]. Even if the exact mechanisms which are responsible for this inflammatory response remain elusive, there is a multitude of malignancies whose growth is enhanced by chronic inflammation [51]. A chronic inflammatory condition by exposure to pathogens such as, e.g., *Helicobacter pylori* in gastric cancer [52] or hepatitis B and C virus in hepatocellular carcinoma [53], can promote carcinogenesis. Moreover, the development of colorectal carcinoma is favored in patients with inflammatory bowel disease [54] but the intake of non-steroidal anti-inflammatory drugs reduces the risk by about 50% [55]. Recently, a TNFα-dependent mechanism was described, which promoted the invasion of OSCC by oral inflammation [56]. Nowadays, tissue remodeling by continuous exposure to immune cells and their mediators can be considered proof that can lead to a tissue-damaging cascade of immunological mechanisms, which can promote the development of neoplastic cells [51]. The compelling evidence between inflammation and enhanced carcinogenesis may also be a valid approach for explaining the correlation between RBC administration and the increased occurrence of M+ found in this study.

## 5. Conclusions

This is the first study to report RBC transfusions as a significant risk factor for development of distant metastasis in OSCC. After thorough consideration of patient-specific features regarding general health conditions and the extent of therapy, confounding variables were identified and a multivariate regression model was created. RBC transfusion and pN stage proved to be independent prognostic factors for the development of M+. OS and TFS were assessed additionally using multivariate analysis. Neither OS nor TFS was significantly influenced by RBC transfusions in the multivariate analysis. Compared to other studies, a similar long-term outcome was found in our cohort. As OSCC patients with M+ face a dire prognosis, all possibilities should be considered to avoid the development of M+ in perioperative management. As the preoperative timeframe from diagnosis until the start of cancer treatment is short, possibilities to optimize patients’ general health condition and preoperative hemoglobin values, e.g., with iron substitution are often limited. However, according to the results of this study, the use of RBC transfusions should be reduced whenever reasonable in the perioperative setting, following the local or national transfusion guidelines.

## Figures and Tables

**Table 1:** Comparison of patient specific parameters of patients with and without blood transfusion.

	No.	Not Transfused	Transfused	*p*-Value
Total	588	436/74.15%	152/28.85%	
Age				
<45	33	23/69.70%	10/30.3%	0.209
45–65	291	208/71.48%	83/28.52%	
>65	264	205/77.65	59/22.35%	
Gender				
Female	254	195/76.77%	59/23.23%	0.217
Male	334	241/72.16%	93/27.84%	
Nicotine				
No	303	240/79.21%	63/20.79%	**0.005**
Yes	285	196/68.77%	89/31.23%	
Alcohol				
No	379	297/78.36%	82/21.64%	**0.002**
Yes	209	139/66.51%	70/33.49%	
pT				
1	271	243/89.67%	28/10.33%	**<0.0001**
2	182	135/74.18%	47/25.82%	
3	62	33/53.23%	29/46.77	
4	73	25/34.25%	48/65.75%	
pN				
1	353	278/78.75%	75/21.25%	**<0.0001**
2	79	49/62.03%	30/37.97	
3	85	48/56.47%	37/43.53%	
4	11	4/36.36%	7/63.64%	
Localization				
1	267	182/68.16%	85/31.84%	**<0.0001**
2	148	129/87.16%	19/12.84%	
3	19	10/52.63%	9/47.37%	
4	48	40/83.33%	8/16.67%	
5	65	54/83.08%	11/16.92%	
6	40	21/52.5%	19/47.5%	
L				
No	387	296/76.49%	91/23.51%	**<0.0001**
Yes	98	55/56.12%	43/43.88%	
V				
No	478	346/72.38%	132/27.62%	0.955
Yes	7	5/71.43%	2/28.57%	
G				
1	74	59/79.73%	15/20.27%	0.187
2	496	284/71.72%	112/28.28%	
3	80	60/75%	20/25%	
4	3	1/33.33%	2/66.67%	
UICC				
I	234	209/89.32%	25/10.68%	**<0.0001**
II	115	91/79.13%	24/20.87%	
III	94	65/69.15%	29/30.85%	
IV	145	71/48.97%	74/51.03%	
Adjuvant Therapy				
no	407	332 81.57%	75/18.43%	**<0.0001**
RTX	134	84/62.69%	50/37.31%	
RCTX	47	20/42.55%	27/57.45%	
Microvascular Transplant				
no	374	343/91.71%	31/8.29%	**<0.0001**
yes	214	93/43.46%	121/56.51%	

Fisher’s exact test was used to examine for statistically significant differences (*p* < 0.05) in dependence of the transfusion status. Localization: 1 = floor of mouth and mandible, 2 = tongue, 3 = oropharynx, 4 = cheek and anterior lip, 5 = maxilla, 6 = multilocular. Adjuvant therapy: no = no adjuvant treatment, RTx = radiation, RCTx = radio- and chemotherapy.

**Table 2:** Univariate Cox regression analysis on the frequency of patient specific factors in correlation to M+, OS and TFS.

	*n* (Total = 588)	M+ (Distant Metastasis)		OS (Overall Survival)		TFS (Tumor-Free Survival)	
		Failures (*n* = 74)	*p*	Failures (*n* = 321)	*p*	Failures (*n* = 241)	*p*
Transfusion		**<0.0001**		**0.0001**		**<0.0001**	
0	436	41/9.40%		248/56.88%		182/41.74%	
1–3	102	23/22.55%		47/46.08%		43/42.16%	
>3	50	10/20%		26/52%		16/32%	
Age		0.3695		**<0.0001**		0.3695	
<45	33	2/6.06%		23/69.7%		18/54.55%	
45–65	291	42/14.43%		173/59.45%		128/43.99%	
>65	264	30/11.36%		125/47.35%		95/35.98%	
Gender		0.9334		**0.0449**		0.9334	
Male	334	41/12.28%		165/49.40%		121/36.23%	
Female	254	33/12.99%		156/61.42%		120/47.24%	
Nicotine		0.4981		0.7823		0.4038	
Yes	285	39/13.68%		151/52.98%		108/37.89%	
No	303	35/11.55%		170/56.11%		133/43.89%	
Alcohol		0.7819		0.0533		0.7745	
Yes	209	24/11.48%		101/48.33%		71/33.97%	
No	379	50/13.19%		220/58.05%		170/44.85%	
Cancer History		0.3946		0.1917		0.6032	
Yes	46	6/13.04%		26/56.52%		21/45.65%	
No	542	68/12.55%		295/54.43%		220/40.59%	
pT		**0.0281**		**<0.0001**		**<0.0001**	
1	271	29/10.7%		161/59.41%		124/45.76%	
2	182	23/12.64%		96/52.75%		71/39.01%	
3	62	10/16.13%		33/53.23%		26/41.94%	
**4**	73	11/16.44%		31/42.47%		20/27.40%	
pN		**<0.0001**		**<0.0001**		**<0.0001**	
0	353	27/7.65%		224/63.46%		167/47.31%	
1	79	19/24.05%		36/45.57%		27/34.18%	
2	85	19/22.35%		32/37.65%		23/27.06%	
3	11	2/18.18%		7/63.64%		6/54.55%	
Localization		0.3261		**0.0001**		**0.0006**	
1	267	35/13.11%		135/50.56%		97/36.33%	
2	148	15/10.14%		100/67.57%		82/55.41%	
3	19	3/15.79%		3/15.79%		2/10.53%	
4	48	7/14.58%		26/54.17%		18/37.50%	
5	65	6/9.23 %		37/56.92%		28/43.08%	
6	40	8/20%		19/47.50%		13/32.50%	
L		**<0.0001**		**<0.0001**		**<0.0001**	
Yes	98	26/26.53%		244/63.05%		34/34.69%	
No	387	98/20.21%		46/46.94%		187/48.32%	
V		**0.0456**		**0.0256**		0.0799	
Yes	7	2/28.57%		2/28.57%		1/14.29%	
No	478	7/1.44%		288/60.25%		220/46.03%	
Pn		**0.0093**		**0.0003**		**0.0019**	
0	438	55/12.56%		269/61.42%		203/46.35%	
1	45	11/24.44%		20/44.44%		17/37.78%	
**G**		**0.0062**		**0.0001**		**0.0032**	
1	74	4/5.41%		51/68.92%		44/59.46%	
2	396	53/13.38%		214/54.04%		151/38.13%	
3	80	15/18.75%		31/38.75%		25/31.25%	
4	3	1/33.33%		1/33.33%		1/33.33%	
UICC		**0.0002**		**<0.0001**		**<0.0001**	
I	234	20/8.55%		145/61.97%		109/46.58%	
II	115	10/8.7%		66/57.39%		51/44.35%	
III	94	17/18.09%		46/48.94%		35/37.23%	
IV	145	27/18.62%		64/44.14%		46/31.72%	
Adjuvant Therapy		**<0.0001**		**<0.0001**		**<0.0001**	
No	407	29/7.13%		239/58.72%		181/44.47%	
RTx	134	22/16.42%		64/47.76%		48/35.82%	
RCTx	47	23/48.94%		18/38.30%		12/25.53%	
Microvascular Transplant		**<0.0001**		**<0.0001**		**<0.0001**	
Yes	214	40/18.69%		94/43.93%		72/33.64%	
No	374	34/9.09%		227/60.70%		169/45.19%	
Preoperative Hemoglobin		**0.0256**		**0.0256**		**0.0002**	
≥12 g/dL	521	63/12.09%		288/55.28%		215/41.27%	
<12 g/dL	67	11/16.42%		33/49.25%		26/38.81%	

A *p*-value < 0.05 indicates a significant correlation between the patient specific factor and the according outcome parameter, which should therefore be considered as a confounding factor. Localization: 1 = floor of mouth and mandible, 2 = tongue, 3 = oropharynx, 4 = cheek and anterior lip, 5 = maxilla, 6 = multilocular. Adjuvant therapy: no = no adjuvant treatment, RTx = radiation, RCTx = radio- and chemotherapy.

**Table 3:** Multivariate Cox regression analysis of the correlation of the transfusion status and the outcome parameters M+, OS, and TFS adjusted for age, gender, pT-, pN-classification, use of microvascular transplant, and preoperative hemoglobin value.

	M+ (Distant Metastasis)				OS (Overall Survival)				TFS (Tumor-Free Survival)			
	HR	SD	95% CI	*p*	HR	SD	95% CI	*p*	HR	SD	95% CI	*p*
Transfusion												
Yes	2.42	0.78	1.28–4.56	**<0.01**	1.11	0.2	0.78–1.57	0.566	1.16	0.22	0.8–1.67	0.437
Age												
High	1.01	0.01	0.99–1.04	0.334	1.03	0.01	1.02–1.04	**<0.01**	1.02	0.01	1.01–1.03	**<0.01**
Gender												
Female	0.73	0.2	0.42–1.26	0.26	1.41	0.21	1.06–1.88	**0.02**	1.07	0.16	0.80–1.44	0.633
pT Stage												
pT2	1.04	0.34	0.55–1.97	0.894	1.21	0.2	0.87–1.68	0.251	1.4	0.24	1.01–1.96	**0.045**
pT3	1.53	0.64	0.67–3.46	0.31	1.68	0.39	1.06–2.66	0.026	2.21	0.54	1.37–3.57	**<0.01**
pT4	0.97	0.41	0.42–2.24	0.952	1.51	0.34	0.97–2.34	0.065	1.97	0.44	1.27–3.1	**<0.01**
pN Stage												
pN1	2.99	0.97	1.59–5.63	**<0.01**	1.36	0.25	0.95–1.95	**0.094**	1.24	0.23	0.85–1.81	0.266
pN ≥ 2	3.37	1.07	1.81–6.29	**<0.01**	2.07	0.35	1.49–2.88	**<0.01**	1.97	0.35	1.39–2.79	**<0.01**
Microvascular Transplant												
yes	1.44	0.47	0.76–2.73	0.262	1.59	0.25	1.17–2.17	**<0.01**	1.28	0.21	0.93–1.77	0.131
Preoperative Hemoglobin												
<12 g/dL	1.21	0.11	1.00–1.45	0.5	0.93	0.04	0.87–1.02	0.122	0.93	0.44	0.85–1.02	0.149

For the development of distant metastasis only the transfusion status and the pN-classification maintained as statistically significant prognosis factors. For OS age, gender, pN-classification, and the use of a microvascular transplant showed to be statistically significant. For TFS the same correlations as in OS could be found, except for gender and the use of a microvascular transplant.

## Data Availability

The data presented in this study are available on request from the corresponding author. The data are not publicly available due to ethical restrictions.
